# Assessments of social vulnerability on laryngeal cancer treatment & prognosis in the US

**DOI:** 10.1038/s41416-025-03056-8

**Published:** 2025-05-15

**Authors:** David J. Fei-Zhang, Camaren M. Cuenca, Angela D. Haskins, Andrew P. Stein, David G. Lott, Urjeet A. Patel, Stephanie S. Smith, Amy L. Dimachkieh, Nosayaba Osazuwa-Peters, Jill N. D’Souza, Anthony M. Sheyn, Jeffrey C. Rastatter, Daniel C. Chelius

**Affiliations:** 1 Northwestern University Feinberg School of Medicine, Chicago, IL USA; 2 Baylor College of Medicine, Houston, TX USA; 3 Department of Otolaryngology-Head and Neck Surgery, Baylor College of Medicine, Houston, TX USA; 4 Department of Otolaryngology-Head and Neck Surgery, Northwestern University Feinberg School of Medicine, Chicago, IL USA; 5 Division of Laryngology, Department of Otolaryngology/Head and Neck Surgery, Mayo Clinic Arizona, Phoenix, AZ USA; 6 Department of Otolaryngology-Head and Neck Surgery, Pediatric Thyroid Tumor Program and Pediatric Head and Neck Tumor Program, Baylor College of Medicine, Texas Children’s Hospital, Houston, TX USA; 7 Department of Head and Neck Surgery & Communication Sciences, Duke University School of Medicine, Durham, NC USA; 8 Department of Population Health Sciences, Duke University School of Medicine, Durham, NC USA; 9 Duke Cancer Institute, Duke University, Durham, NC USA; 10 Louisiana State University Health Sciences Center Department of Otolaryngology and Division of Pediatric Otolaryngology Children’s Hospital of New Orleans, New Orleans, LA USA; 11 Department of Pediatric Otolaryngology, Le Bonheur Children’s Hospital, Memphis, TN USA; 12 Department of Otolaryngology-Head and Neck Surgery, University of Tennessee Health Science Center, Memphis, TN USA; 13 Department of Pediatric Otolaryngology, St. Jude Children’s Research Hospital, Memphis, TN USA; 14 Division of Pediatric Otolaryngology, Ann & Robert H. Lurie Children’s Hospital of Chicago, Chicago, IL USA

**Keywords:** Epidemiology, Social sciences, Cancer epidemiology, Head and neck cancer, Risk factors

## Abstract

**Background:**

Previous social determinants of health (SDoH) studies on laryngeal cancer (LC) have assessed individual factors of socioeconomic status and race/ethnicity but seldom investigate a wider breadth of SDoH-factors for their effects in the real-world. This study aims to delineate how a wider array of SDoH-vulnerabilities interactively associates with LC-disparities.

**Methods:**

This retrospective cohort study assessed 74,495 LC-patients between 1975 and 2017 from the Surveillance-Epidemiology-End Results (SEER) database using the Social Vulnerability Index (SVI) from the CDC, total SDoH-vulnerability from 15 SDoH variables across specific vulnerabilities of socioeconomic status, minority-language status, household composition, and infrastructure/housing and transportation, which were measured across US counties. Univariate linear and logistic regressions were performed on length of care/follow-up and survival, staging, and treatment across SVI scores.

**Results:**

Survival time dropped significantly by 34.37% (from 72.83 to 47.80 months), and surveillance time decreased by 28.09% (from 80.99 to 58.24 months) with increasing overall social vulnerability, alongside advanced staging (OR 1.15; 95%CI 1.13–1.16), increased chemotherapy (OR 1.13; 95%CI 1.11–1.14), decreased surgical resection (OR 0.91; 95%CI 0.90–0.92), and decreased radiotherapy (OR 0.97; 95%CI 0.96–0.99).

**Discussion:**

In this SDoH-study of LCs, detrimental care and prognostic trends were observed with increasing overall SDoH-vulnerability.

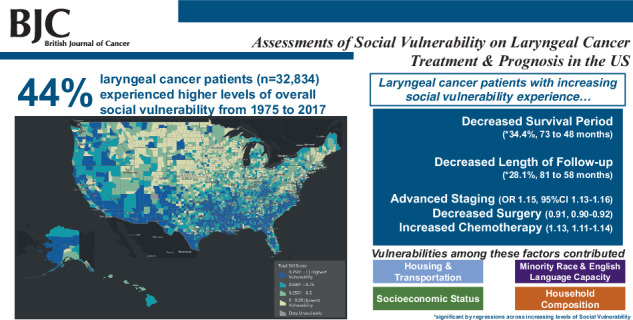

## Introduction

Laryngeal cancer accounted for nearly 19% of all new head and neck cancer diagnoses in the United States in 2023 [1]. While the incidence of laryngeal cancer has decreased over the past 30 years, overall mortality rates have increased, largely contributed by patients being diagnosed with late-staged malignancies on first presentation [2]. Thus, many investigations have sought to identify what clinicodemographic factors have contributed to this increased risk for morbidity and mortality related to laryngeal cancer.

Social determinants of health (SDoH) have been increasingly investigated in relation to the disparities found within head and neck cancers (HNC) in adult populations. Studies have found that patient race and ethnicity, socioeconomic status (SES), sex, insurance status, and rurality, each account for disparities in survival of HNC [3–5]. Regarding laryngeal cancer, Liu et al. [6] observed that patients with higher SES had better survival, but Black patients with high SES had significantly decreased survival rates compared to White patients. Shaikh et al. [7] found that being a member of a racial or ethnic minority, advanced age, female sex, residing more than 30 miles from treatment a facility, and lack of insurance, was associated with greater diagnosis-to-treatment interval. However, given that the influences of SDoH are simultaneously experienced and derived from a patient’s lived-in environment, prior investigations have yet to encapsulate this amalgamated impact of these represented factors and others, downplaying the actual apparent disparities imparted by SDoH, especially in regards to SDoH-vulnerabilities on a community-level. Thus, comprehensive approaches to assess this interplay of a larger scope of SDoH and their associations with disparities in laryngeal cancer remains in dire need.

The Social Vulnerability Index (SVI) is a tool developed by the US Centers for Disease Control and Prevention based on US Census data featuring 15 SDoH factors grouped into 4 themes: SES, household composition, minority race/ethnicity and English language status, and housing type & transportation [8]. These themes are ranked along every census tract and county, creating a nation-wide framework by which clinicians can quantifiably evaluate social vulnerability influence and their relation to health disparities. The SVI has been utilized previously to evaluate the interaction between multiple SDoH and their impact on care and survival in pediatric HNC and head and neck melanomas [9, 10]. Given the gaps in understanding SDoH-influences on laryngeal cancer disparities, our study aims to apply SVI towards a national patient cohort to analyze the relationships of summated and specific SDoH-vulnerabilities with the care and prognostic outcomes of adult laryngeal squamous cell cancer patients in the United States.

## Methods

This retrospective cohort study follows the Strengthening the Reporting of Observational Studies in Epidemiology (STROBE) reporting guideline. The Northwestern University IRB/ethics committee has exempted this study (STU00216871) due to the data queried consisting of publicly available, de-identified data.

### Databases

The CDC-SVI was queried for ranked scores among 15 census factors within four SDoH themes of socioeconomic status (poverty, unemployment, income level, high school, diploma status), minority status-language (minority status, proficiency with English), household composition (household members 65+ years, household members ≤ 17 years, disability status, single-parent status), and housing-transportation (multiunit structure, mobile homes, crowding, no vehicle, group quarters), as well as total composite scores. Based on CDC-SVI documentation, SVI-theme sub-scores are differentially weighed to formulate the total composite score and are assigned different weights based on sociodemographic-census data of the designated area. Total and SVI-theme scores are based on relative social vulnerabilities of a particular census tract among all 72,158 US census tracts, ranging from 0 to 1, with 0 representing the lowest social vulnerability and 1 representing the highest [8]. Further description of these formulations can be found in the Methods Supplement.

The National Cancer Institute-Surveillance, Epidemiology, and End Results Program (NCI-SEER) database contains national datasets of patient variables, pathological characteristics, treat- ment modalities, and prognostic outcomes. Months under surveillance represent a length-of-care measurement reflecting the active follow-up a patient receives for their primary malignancy up until the last provider interaction. Months survival represents active follow-up until patient suffers a mortal outcome.

SVI scores were abstracted and matched to SEER-patient data based on county of residence at the time of diagnosis. County-assigned scores were generated by weighted score means per population density of each census tract within the county. These methodologies were adapted from prior SVI-based investigations for heterogenous database linkage [9, 10]. A schematic workflow of this data linkage process can be found in Supplement eFigure 1. These are also fully described in the Methods Supplement.

### Population definitions

SEER was queried for adult (20+ years) patients diagnosed with laryngeal squamous cell carcinomas between 1975 and 2017. Head–neck regions were extracted using International Classification of Diseases for Oncology, Third Edition (ICD-O-3) topographic codes [C32.0-C32.9].

### Statistical analysis

Months surveillance or followed up within each disease class were analyzed by total CDC-SVI score and theme subscores. CDC-SVI scores were split into relative equivalently sampled quintiles based on actual scores within each disease class. The relative SVI quintiles were delineated by quintiles of gradually increasing percentile based on their actual SVI-scores (i.e. “less than 20’, “20 to 39.99”, “40 to 59.99”, “60 to 79.99”, and “80 to 99.99”) per disease class (e.g., within disease A, patients with the lowest CDC-SVI scores are grouped into the “less than 20” quintile group, then the “20 to 39.99” represents the next set of higher scores, and etc.).

Among these total and SVI-theme quintiles, differences between the mean surveillance months for lowest and highest SVI- scored quintiles were calculated. Trend significance was assessed by linear regression across all data points against relative-SVI quintiles for surveillance months (i.e., not a trend through the base- line descriptive values), and violin plots were generated to assess relative sample distribution for surveillance months along the contour widths within each relative-SVI quintile while simultaneously measuring the median, interquartile range (IQR), and 1.5 times the IQR with its inner box plot. Means, standard deviations, and ranges for surveillance months per quintile were also calculated. The proportion of patients who were alive/lost to follow-up or dead was calculated per quintile.

Survival months were analyzed similarly as surveillance months. However, patients who were alive/lost upon last follow-up were excluded to extract patients who were dead upon last follow-up. Primary surgery, chemotherapy, and radiation (external beam) therapy occurrence and advanced staging on time of diagnosis within disease classes were analyzed with univariate logistic regression across relative SVI quintiles per CDC-SVI category (reference being least socially vulnerable/”<20” quintile, compared to increasing quintiles of vulnerability). Univariate analyses, rather than multivariate, were selected due to the preservation of differential weights applied to each of the SVI-themes in formulating the total/overall SVI scores for each region. Full description of this rationale.

Statistical significance was set as *p*-value < 0.05. Two-sided p-values were reported for analyses.

## Results

There were 74,495 patients with laryngeal squamous cell neoplasms identified in the SEER database and included in the analytic cohort. The most represented clinicodemographic characteristics were patients aged 65–84 years (*n* = 34,651, 46.5%), being of male sex (*n* = 60,114, 80.7%), non-Hispanic white race/ethnicity (*n* = 56,606, 76.0%). Primary sites of glottic (*n* = 39,779, 53.4%), supraglottic (*n* = 25,672, 34.5%), and subglottic (*n* = 1129, 1.5%) were well-represented. Total SVI scores ranged from 0.000 to 0.947. SES SVI scores ranged from 0.000 to 0.976. Minority-language (ML) SVI scores ranged from 0.002 to 0.945. Household-composition (HC) SVI scores ranged from 0.091 to 0.971. Housing-transportation (HT) SVI scores ranged from 0.051 to 0.942.

Further patient clinical characteristics stratified by total CDC-SVI scores are summarized in Table 1.

**Table 1:** Patient Characteristics with Increasing Total Social Vulnerability.

Characteristic	*N*	Total SVI Score				
		0.000–0.199, *N* = 13314 (18%)	0.200–0.399, *N* = 15626 (21%)	0.400–0.599, *N* = 12721 (17%)	0.600–0.799, *N* = 17209 (23%)	0.800–0.999, *N* = 15625 (21%)
Age	74,495					
20-44 years		467 (3.5%)	595 (3.8%)	465 (3.7%)	685 (4.0%)	482 (3.1%)
45-64 years		6066 (46%)	7124 (46%)	5673 (45%)	8405 (49%)	7122 (46%)
65-84 years		6257 (47%)	7378 (47%)	6044 (48%)	7539 (44%)	7433 (48%)
85+ years		524 (3.9%)	529 (3.4%)	539 (4.2%)	580 (3.4%)	588 (3.8%)
Sex	74,495					
Male		10,711 (80%)	12,597 (81%)	10,409 (82%)	13,752 (80%)	12,645 (81%)
Female		2603 (20%)	3029 (19%)	2312 (18%)	3457 (20%)	2980 (19%)
Race	74,495					
White		12,178 (91%)	13,101 (84%)	9225 (73%)	11,731 (68%)	10,371 (66%)
Black		625 (4.7%)	1669 (11%)	1526 (12%)	4198 (24%)	2609 (17%)
Hispanic		352 (2.6%)	520 (3.3%)	860 (6.8%)	833 (4.8%)	2058 (13%)
Asian or Pacific Islander		101 (0.8%)	258 (1.7%)	1,019 (8.0%)	341 (2.0%)	482 (3.1%)
Native American		24 (0.2%)	44 (0.3%)	46 (0.4%)	65 (0.4%)	51 (0.3%)
Unknown		34 (0.3%)	34 (0.2%)	45 (0.4%)	41 (0.2%)	54 (0.3%)
Region	74,495					
Midwest		4274 (32%)	3562 (23%)	705 (5.5%)	5,940 (35%)	90 (0.6%)
Northeast		5694 (43%)	4562 (29%)	763 (6.0%)	1,053 (6.1%)	497 (3.2%)
South		1633 (12%)	2549 (16%)	2257 (18%)	5226 (30%)	5481 (35%)
West		1713 (13%)	4953 (32%)	8996 (71%)	4990 (29%)	9557 (61%)
Primary Site	74,495					
Larynx Glottic		7200 (54%)	8363 (54%)	7126 (56%)	8781 (51%)	8309 (53%)
Larynx Other		1364 (10%)	1613 (10%)	1144 (9.0%)	2035 (12%)	1,759 (11%)
Larynx Subglottic		204 (1.5%)	221 (1.4%)	184 (1.4%)	253 (1.5%)	267 (1.7%)
Larynx Supraglottic		4546 (34%)	5429 (35%)	4267 (34%)	6140 (36%)	5290 (34%)
TNM/AJCC Combined Stage	71,572					
Stage I-III		10,561 (84%)	12,689 (84%)	10,059 (82%)	13,093 (79%)	11,544 (77%)
Stage IV & Above		2069 (16%)	2332 (16%)	2199 (18%)	3519 (21%)	3507 (23%)
SEER-desginated Grade	58,748					
Grade I or II		8082 (77%)	9425 (78%)	7725 (77%)	10,114 (76%)	9761 (76%)
Grade III or above		2410 (23%)	2701 (22%)	2341 (23%)	3186 (24%)	3003 (24%)
Primary Surgery Performed	72,640					
No Surgery		6,446 (51%)	7479 (50%)	6984 (56%)	9386 (55%)	9142 (59%)
Surgery		6,285 (49%)	7448 (50%)	5597 (44%)	7596 (45%)	6277 (41%)
Radiation Therapy Performed	74,495					
No Therapy		3694 (28%)	4005 (26%)	3112 (24%)	4707 (27%)	4513 (29%)
Therapy		9620 (72%)	11,621 (74%)	9609 (76%)	12,502 (73%)	11,112 (71%)
Chemotherapy Performed	74,495					
No Therapy		10,701 (80%)	12,805 (82%)	10,279 (81%)	12,972 (75%)	11,689 (75%)
Therapy		2613 (20%)	2821 (18%)	2442 (19%)	4237 (25%)	3936 (25%)
Vital Status on Last Follow-up	74,495					
Alive		3989 (30%)	3795 (24%)	3767 (30%)	5348 (31%)	5465 (35%)
Dead		9325 (70%)	11,831 (76%)	8954 (70%)	11,861 (69%)	10,160 (65%)

### Trends in survival months with increasing social vulnerability

**Fig. 1:**
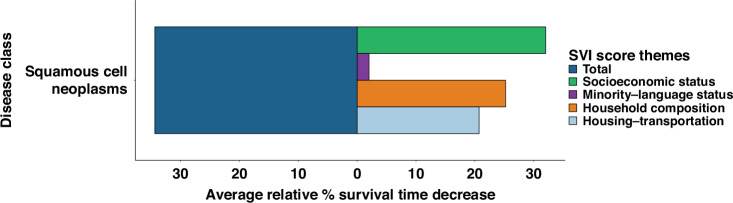
Relative decreases in months survival with increasing SVI scores. Percentage decreases from lowest to highest-SVI quintiles based on mean months survived for total-SVI score and subcomponent SVI-theme subscores.

With increased total SVI score, significant decreases in mean months survival among patients with laryngeal cancer were observed. Significant differences in mean months survival between the lowest and highest total SVI quintile was 34.37% (72.83 to 47.80 months; *p* < 0.001) (Fig. 1, Supplement eFigure 2). By magnitude, SES vulnerabilities were the greatest contributors to total vulnerability trends, followed by HC, HT, then ML status (Fig. 1).

### Trends in months under surveillance with increasing social vulnerability

**Fig. 2:**
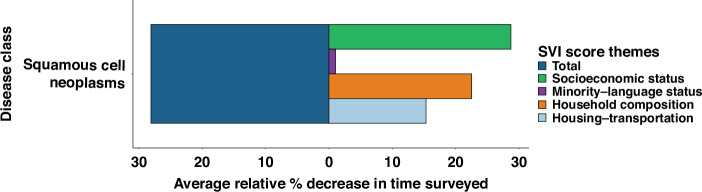
Relative decreases in surveillance months with increasing SVI scores. Percentage decreases from lowest to highest-SVI quintiles based on mean surveillance months for total-SVI score and subcomponent SVI-theme subscores.

Mirroring the trends seen in survival, there was a significant decrease in months of surveillance/follow-up with increasing total SVI score. Between the first and fifth SVI quintile, surveillance time decreased 28.09% (80.99 to 58.24 months; *p* < 0.001) (Fig. 2, Supplement eFigure 3. Across SVI trends, SES vulnerabilities contributed the most to total vulnerability trends, followed by HC and HT (Fig. 2).

### Advanced staging and high grading on presentation occurrence with increasing social vulnerability

Increasing total SVI score was associated with increased odds of advanced staging (OR 1.15; 95% CI, 1.13–1.16, *p* < 0.001) and high grading (OR 1.02. 95%CI 1.01–1.03; *p* = 0.029). For advanced staging, SES-vulnerability was most associated with this overall trend, followed by HC and HT. For high grade, HT was most associated with this overall trend (Table 2).

**Table 2:** Late-Staging and High-Grading on Preliminary Presentation with Increasing SVI Scores.

Characteristic	Advanced Stage^1^			High Grade		
	OR	95% CI	*p*-value	OR	95% CI	*p*-value
Total	1.15	1.13, 1.16	< 0.001	1.02	1.01, 1.03	0.029
Socioeconomic Status	1.17	1.16, 1.19	< 0.001	1.01	1.00, 1.02	0.199
Minority-Language Status	1.00	0.98, 1.01	0.706	1.01	1.00, 1.03	0.095
Household Composition	1.11	1.09, 1.12	< 0.001	1.01	0.99, 1.02	0.403
Housing-Transportation	1.06	1.04, 1.07	< 0.001	1.02	1.01, 1.03	0.019

^1^By American Joint Committee on Cancer, 6th Edition (AJCC-6).

Univariate logistic regressions across SVI quintiles based on first presentation occurrence of Stage IV/distant expansion for increasing total-SVI score and subcomponent SVI-theme subscores per disease class.

### Treatment receipt with increasing social vulnerability

Patients with laryngeal cancers displayed significantly increased odds of receiving chemotherapy with increased total SVI score (OR 1.13; 95% CI, 1.11–1.14; *p* < 0.001). Increasing SES, followed by HC-vulnerability contributed to these overall social vulnerability trends of chemotherapy receipt (Table 3).

**Table 3:** Treatment Receipt with Increasing SVI scores.

Treatment Type	Characteristic	OR	95% CI	*p*-value
Chemotherapy	Total	1.13	1.11, 1.14	< 0.001
	Socioeconomic Status	1.18	1.17, 1.20	< 0.001
	Minority-Language Status	0.96	0.95, 0.97	< 0.001
	Household Composition	1.12	1.10, 1.13	< 0.001
	Housing-Transportation	1.00	0.99, 1.02	0.448
Radiation Therapy	Total	0.97	0.96, 0.99	< 0.001
	Socioeconomic Status	0.97	0.96, 0.98	< 0.001
	Minority-Language Status	1.00	0.99, 1.01	0.597
	Household Composition	1.00	0.98, 1.01	0.422
	Housing-Transportation	1.00	0.99, 1.01	0.996
Surgical Resection	Total	0.97	0.96, 0.99	< 0.001
	Socioeconomic Status	0.97	0.96, 0.98	< .001
	Minority-Language Status	1.00	0.99, 1.01	0.597
	Household Composition	1.00	0.98, 1.01	0.422
	Housing-Transportation	1.00	0.99, 1.01	0.996

Univariate logistic regressions across SVI quintiles based on primary surgical resection, radiation therapy, and chemotherapy receipt with increasing total-SVI score and subcomponent SVI-theme vulnerabilities.

Increases in total social vulnerability were associated with significantly decreased odds of a patient receiving radiation therapy (OR 0.97; 95% CI, 0.96–0.99; *p* < 0.001). SES vulnerability contributed significantly to these overall social vulnerability trends (Table 3).

Increasing total SVI scores were associated with significantly decreased odds of having surgical resection performed (OR 0.91; 95% CI, 0.90–0.92; *p* < 0.001). SES vulnerability contributed significantly to these overall social vulnerability trends (Table 3).

## Discussion

To our current knowledge, this is the first study to utilize the SVI comprehensively in order to evaluate the impact of SDoH in a nationally-representative patient population diagnosed with laryngeal squamous cell carcinomas while accounting for the differential influence of multiple social vulnerability factors. Overall, increasing social vulnerability were associated with decreases in months under surveillance and survival, increased odds of advanced staging at presentation, and decreased odds of radiation therapy or primary surgery receipt.

As demonstrated by our group’s prior work, the SVI themes offer a distinctive interpretation of the interplay between SDoH factors, influencing the associations between social vulnerability and laryngeal cancer outcomes [9, 10]. These methods allow for showcasing how the culmination of multiple social vulnerabilities impact patient outcomes while simultaneously elaborating on the interactions of each SDoH theme on one another. Instead of utilizing individual-level variables of SDoH, the SVI factors in the sociodemographic contexts of each census tract by differentially weighing each theme sub-score to calculate total social vulnerability. This adaptable feature of the SVI allowed for our utilization of non-multivariable regression analyses, as performing such multivariate analyses would result in many of the SVI associations and their outcomes without their sociodemographic contexts properly represented. Even in comparison to other large data SDoH-indices, such as the Yost Index or Area Deprivation Index, the SVI’s features in delineating separate themes of varied SDoH-factors remain unique [11–13].

The results of our study align with many of those that examined the impact of singular social vulnerabilities on patients with laryngeal cancer. For instance, previous findings that members of racial and ethnic minority groups present with further advanced stages of disease, experience differences in treatment modalities as compared to white patients, and have decreased overall survival conferred with our own [14–21]. However, our study did contrast previous findings with regard to minority group status and its association with advanced initial disease staging [22–24]. Specifically, Shin et al. [22] observed that black and Hispanic patients in the SEER database were diagnosed with advanced stage disease significantly more often than white patients were. Our group found that the ML sub-group was the only theme not associated with increased odds of advanced staging at presentation. This poses the question of whether the advanced staging of disease attributed to race was not due to confounding SDoH variables. Nevertheless, while previous studies explain the individual impact of minority status, ours demonstrates that its impact across various clinical outcomes is still significant in the context of multiple social vulnerabilities. Our findings did not differ from previous studies affirming the associations between SES and education with laryngeal cancer prognosis, as covered in the SES theme score [15, 25].

Considering the detrimental mortality trends of laryngeal cancer seen in this study and overall in the US, our study provides a means of identifying the sociodemographic risk factors patients face pertaining to decreased survival and surveillance time. It is well documented that laryngeal neoplasms present later due to poor screening methods [26]. Aligned with this pattern, this study shows that social vulnerability associates with these disparate outcomes and provides an argument of how these factors should be taken into account when evaluating a patient’s risk for laryngeal cancer, especially in regards to disease management. Given the differences in therapy receipt related to social vulnerabilities, which have been shown to be a point of health disparity in patients with laryngeal cancer [17, 22]. Although our study did not specifically examine the use of laryngeal preservation therapies, we demonstrated that increasing social vulnerability was associated with decreased odds of radiation, a hallmark of salvage therapy [27]. By accounting for the complex interactions of SDoH, our use of the SVI can help inform health professionals of which social vulnerabilities need more investigation and guidance on how they can be used to inform policy and guidelines in managing complex and aggressive diseases like laryngeal cancer.

By utilizing large data and interactive SDoH approaches, modeling of sociodemographic factors in health disparities more accurately mirrors the real-world impact of manifold sociodemographic factors. With such progress, the question becomes less of *what* the problems are and more of *how* to take action against these specifically quantified targets [6, 28, 29]. As indicated by the degree of systemic factors affecting health outcomes, disparities affecting communities largely originate from public, private, and other institutions that make up the surroundings of patients. With these systemic origins, ameliorating community-based SDoH requires reshaping policies across the clinical and non-clinical spectrum, whether that includes neighborhood redistricting, increased subsidies of transportation and health insurance, publicly supported construction of food accessibility and affordable shelter, private-organization outreach, lobbying, and other initiatives [30, 31]. However, as national policy has begun to adopt approaches of addressing laryngeal cancer and other oncologic needs on a large scale, nuanced approaches and discourse of investigations like ours will enable careful resource allocation towards specific SDoH that present the most demonstrated need for support [32]. Next steps necessitate a calculated approach to initiating prospective implementations from large-data retrospective data in local contexts to provide proof of concept for nation-wide initiatives.

### Strengths and Limitations

The foremost strength of this study lies in the use of the SVI to assess a wide range of social vulnerabilities that provide both a breadth of comparative SDoH-measures and the depth of county-level specificity. Furthermore, it also provides quantifiable associative measures of each SDoH-theme for its contributions to overall vulnerability impact on patient outcomes. Other strengths include the large sample size made possible by the utilization of a national database.

However, certain limitations must be considered for this study. Clinicodemographic variables of interest were either unavailable or had substantial levels of missingness, leading to prohibition of further analyses the specific SEER dataset iteration. These include the lack of information into the type of surgical procedure and revisions performed, immunotherapy receipt, and cause-of-death missingness. In light of these shortcomings, utilization of proprietary clinical datasets that require paid-access with fuller cataloging of such detailed measures not all SDoHs are covered by the SVI variables, leaving unknown factors that could contribute to a patient’s overall vulnerability. These restrictions also apply to the SVI, which remains restricted to its 15 SDoH-variables of interest. Future studies should consider the creation of custom SDoH-indices that encompass SVI-related factors, as well as others not traditionally investigated. In addition, considerations of multilevel analyses encompassing individual- and community-level SDoH-factors should be incorporated in future studies to extend beyond this investigation’s largely community-level contexts, as more recent investigations have engaged with [11, 12]. Lastly, as with any large retrospective study, the findings purported here can only be inferred as associative rather than causative for the relationships observed among laryngeal cancer disparities.

## Conclusion

This investigation provides comprehensive insights into how a wide variety of social vulnerabilities interact and influence laryngeal cancer prognosis and treatment disparities within the United States. Not only do our results remain consistent with prior SDoH-studies, they also dynamically contextualize prior findings with socioeconomic status and minority race/ethnicity through additional factor considerations within these themes as well as themes of housing, transportation, and household composition. Through incorporating a large-data, interactional approach of publicly available tools, our insights harken the growing public and private consortium vowing to ameliorate observed nationwide disparities of cancers. Only through the synergies of social disparities observed here can individuals, their local communities, and representative institutions cooperatively initiate the specific prospective investigations and policies to enact positive change for laryngeal cancer disparities.

## Supplementary information

Supplement Figure 1

Supplement Figure 2

Supplement Figure 3

Methods Supplement

## Data Availability

The datasets generated during and/or analysed during the current study are available in the NCI-SEER repository, https://seer.cancer.gov/, and CDC-SVI repository, https://www.atsdr.cdc.gov/place-health/php/svi/index.html.
