# Supplementary figures and images for: Assessments of social vulnerability on laryngeal cancer treatment & prognosis in the US
Source: Br J Cancer. 2025 May 15;133(2):248–54. doi: 10.1038/s41416-025-03056-8 (PMC12304200; doi:10.1038/s41416-025-03056-8)

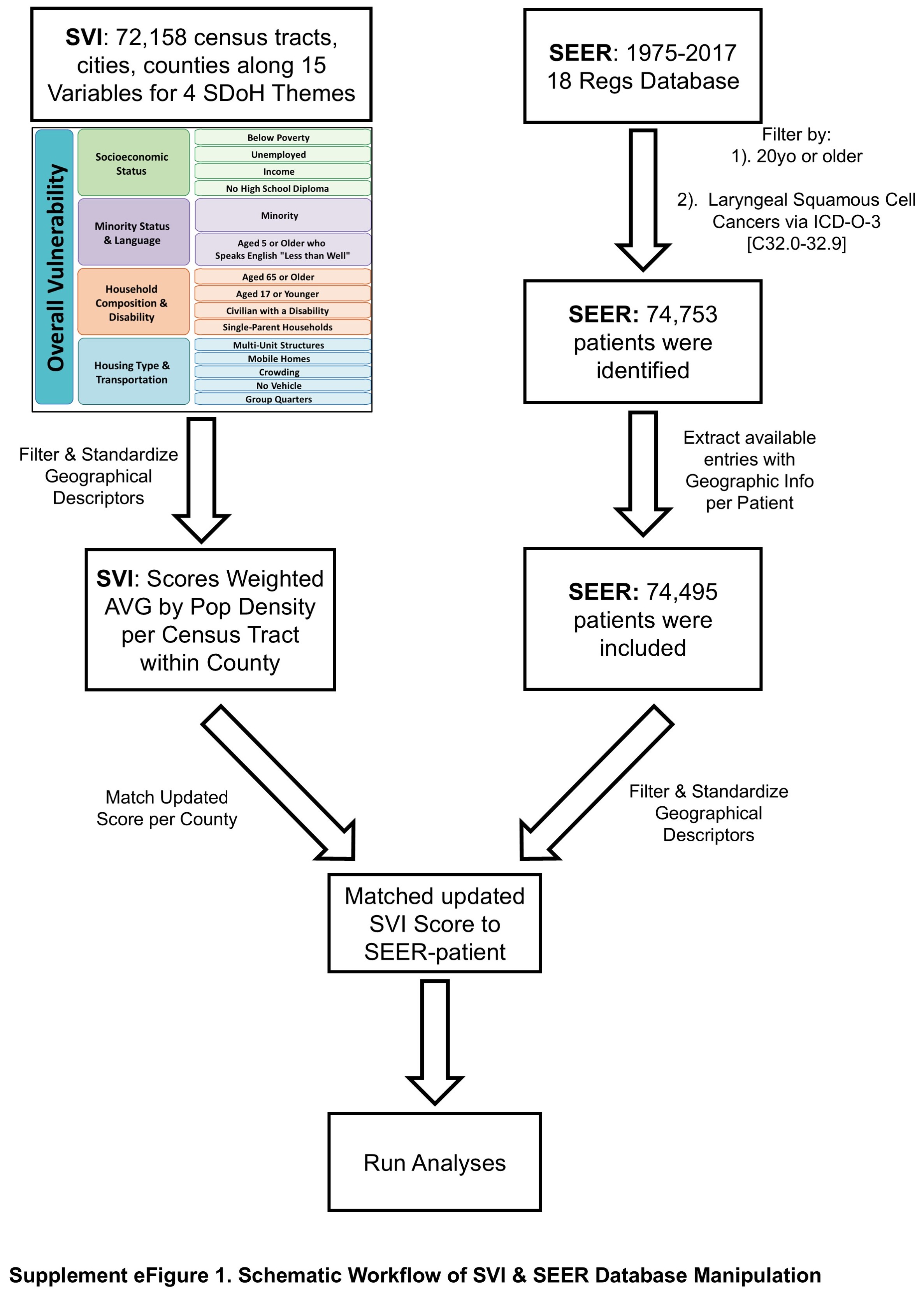

Supplement: Supplementary file 1 — Supplement Figure 1 [file 41416_2025_3056_MOESM1_ESM.jpg]

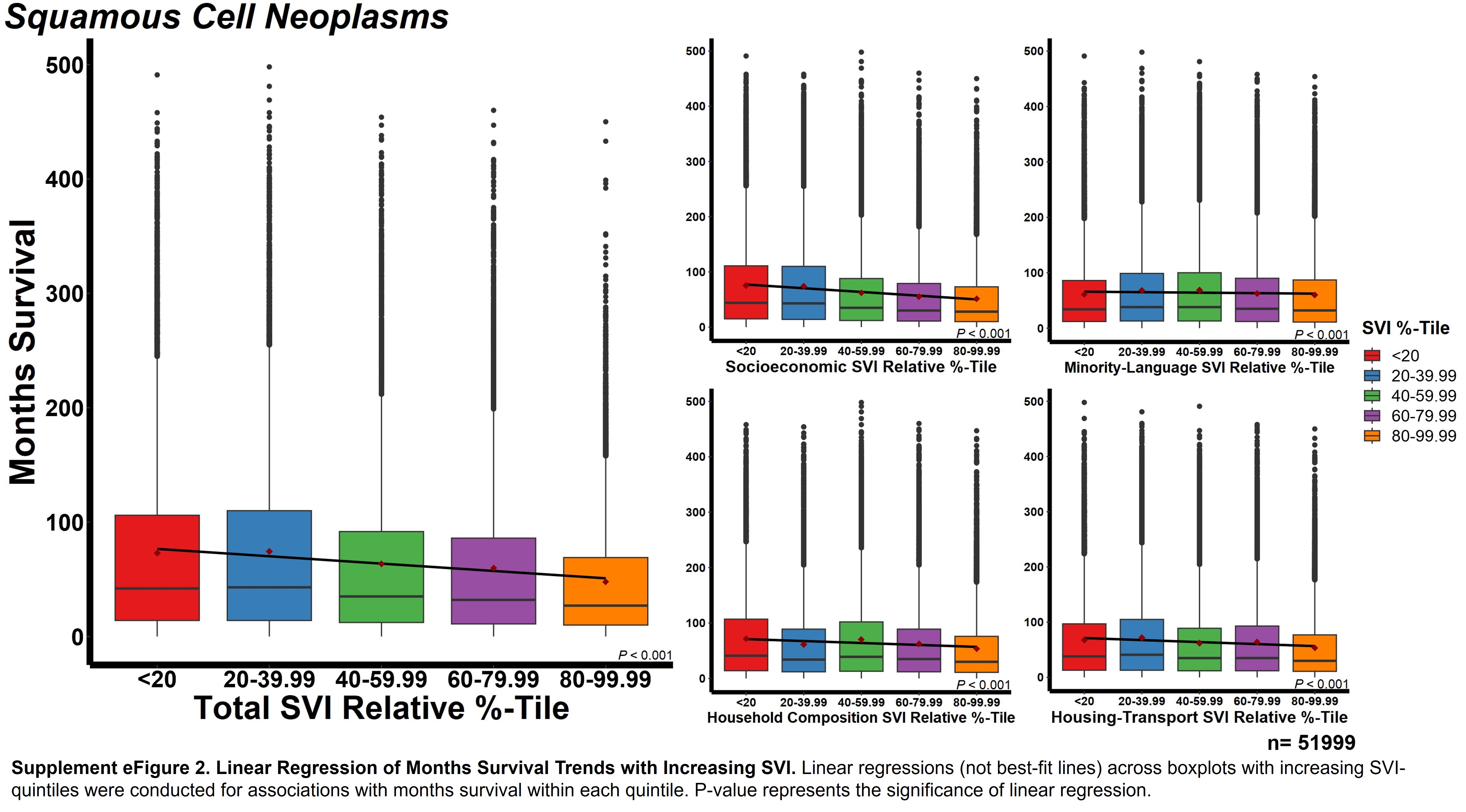

Supplement: Supplementary file 2 — Supplement Figure 2 [file 41416_2025_3056_MOESM2_ESM.jpg]

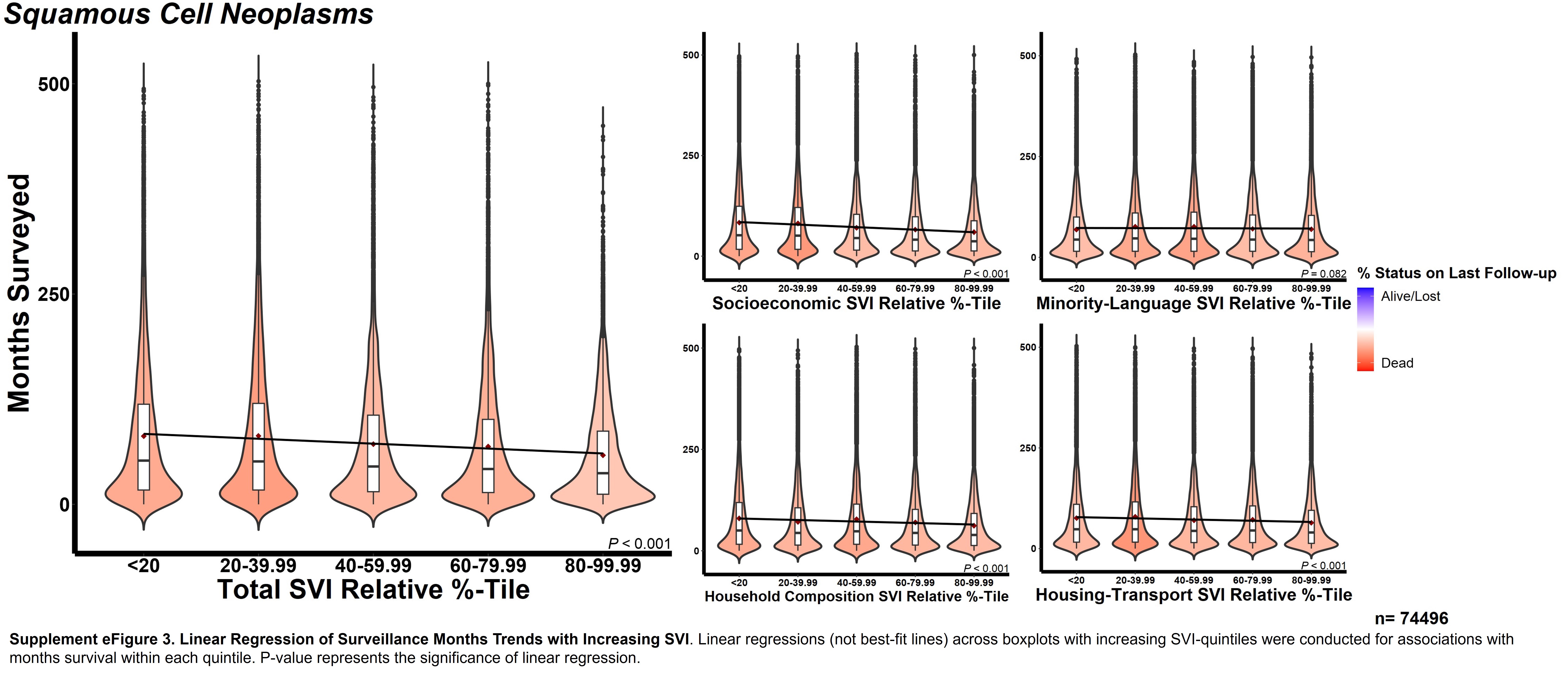

Supplement: Supplementary file 3 — Supplement Figure 3 [file 41416_2025_3056_MOESM3_ESM.jpg]
