# Supplementary material for: Assessments of social vulnerability on laryngeal cancer treatment & prognosis in the US
Source: Br J Cancer. 2025 May 15;133(2):248–54. doi: 10.1038/s41416-025-03056-8 (PMC12304200; doi:10.1038/s41416-025-03056-8)
Supplement: Supplementary file 4 — Methods Supplement [file 41416_2025_3056_MOESM4_ESM.docx]

**Methods and Statistical Analysis Supplement**

Per the methodologies described by the CDC-SVI development team, the 15 social factors are grouped into the 4 SDH themes of SES (poverty, unemployment, income level, and high school diploma status), ML (minoritized racial and ethnic group [American Indian and Alaska Native, Asian, Black or African American, American Indian and Alaska Native, Asian, Native Hawaiian or other Pacific Islander, other, and multiracial (per the US Census) and proficiency with English), HH (household members aged ≥65 and ≤17 years, disability status, single-parent status), and HT (multiunit structure, mobile homes, crowding, no vehicle, group quarters) were assigned weighted-average scores based on the relative proportional makeup of the census populations. These themed scores were ascribed from a range of 0 to 1, representing the relative comparisons across the whole range of standardized census tract areas (e.g., a score of 0.7 represents a census tract being more socially vulnerable in a specific SDH category than 70% of census tracts in the country). A total composite from each of these four themes encompasses a dynamic, differentially weighted average based on internal modeling schemes of sociodemographic contextualization (e.g. Census Tract A could have its Total SVI score calculated based on 35% of the SES-theme, 15% of the ML-theme, 23% of HH-theme, 27% of HT-theme; Census Tract B could be comprised of 33%, 17%, 22%, 28%, respectively; Census Tract C could be 25%, 25%, 25%, 25%, respectively; and etc.) that are not made publicly available. Based on these formulaic manipulations, the total SVI scores also range from 0 to 1 and represent a similar interpretation of the SVI-theme scores (e.g., a score of 0.6 represents a census tract being more socially vulnerable across the total composite of SDH-themes than 60% of census tracts in the country). In order to upcode the categorizations of census tracts to counties, for which census tracts are designated to not overlap into more than one county classification (e.g., Census tracts A, B, C only belong to County A and **cannot** be a part of Counties B, C, etc.), scores that are assigned to the level of counties comprise the population-weighted averages of all the census tracts comprising a specific county. These scores also remain ranged from 0 to 1 and hold similar interpretations as prior census tracts for the total SVI composite and SVI-themes. Upcoding to county-level scores were necessitated due to the level of geocode/geography available within the selected SEER dataset in order to remain compliant with HIPAA and data use agreement standards set by the SEER administrators.

Based on the total SVI’s representation as a dynamic, differentially weighted composite encompassing these themes, univariate regressions were elected to allow interpretations of the total SVI to preserve its real-world contextualized, dynamic differential-weights and, given its summated and composited formulation, retain a multivariate-interpretation rather than a multivariate analysis in concert with the SVI-theme factors (i.e. univariate model of dependent variate outcome and independent variate Total vs. multivariate model of dependent variate outcome, independent covariates of Total SVI, SES, ML, HH, and HT). In other words, the multivariate approach would reassign weights of 20% per category and remove the real-world valences already built into the total SVI measure. Univariate modeling for each of the SVI-themes is also justified by allowing independent delineation of how much certain SDH-themes influence the associations of the total SVI composite by isolating its true value association without confounding by the dynamic weights associated with total SVI calculation (e.g., if Census Tract A has a weight of 20% for ML but Census Tract B has a weight of 30% for ML, we would not know how much ML in isolation confers its influence on either Census Tract’s Total SVI/overall SDH-composite effects in multivariate analyses because of the proprietary/inaccessible nature of the dynamic weights of the total SVI by the CDC).

In re-normalizing the extent of SVI effects to the pertinent patient population, total and subtheme SVI scores of each patient cohort were split into relative equivalently sampled quintiles on a per-histology/subtype level. This allows relevancy of SDH-range to the patient population at hand in the specific comparisons outlined in this investigation’s design (in other words, how does SDH-vulnerability vary within patient populations and what is their influence relative to the span of patient clinicodemographics represented). Quintiles were ranked as discrete variate levels (with reference level set to the lowest vulnerability quintile) within univariate logistic regression models and utilized to assess for associated occurrences of whether patients received primary surgical and/or non-surgical treatment, as well as whether they had advanced staging on preliminary presentation based on SEER-staging/AJCC-TNM-staging classifications available in the selected SEER dataset. Statistical significance was set to P<.05. Two-sided P values were reported for analyses. Analyses were conducted in R version 4.2.3 (R Project for Statistical Computing).
